# Differences in Cerebral Oxygenation in Cardiogenic and Respiratory Cardiac Arrest Before, During, and After Cardiopulmonary Resuscitation

**DOI:** 10.3390/jcm12082923

**Published:** 2023-04-18

**Authors:** Yasuaki Koyama, Akira Ouchi, Nobutake Shimojo, Yoshiaki Inoue

**Affiliations:** 1 Department of Emergency and Critical Care Medicine, Hitachi General Hospital, 2-1-1, Jonan-cho, Hitachi 317-0077, Ibaraki, Japan; 2 Department of Adult Health Nursing, College of Nursing, Ibaraki Christian University, 6-11-1 Omika, Hitachi 319-1295, Ibaraki, Japan; 3 Department of Emergency and Critical Care Medicine, University of Tsukuba Hospital, 2-1-1 Amakubo, Tsukuba 305-8576, Ibaraki, Japan

**Keywords:** near-infrared spectroscopy, cerebral oxygenation, cerebral oxygen saturation, cardiogenic cardiac arrest, respiratory cardiac arrest, hypoxia

## Abstract

We compared the changes in cerebral oxygen saturation (ScO_2_) levels during cardiac arrest (CA) events using porcine models of ventricular fibrillation CA (VF-CA) and asphyxial CA (A-CA). Twenty female pigs were randomly divided into VF-CA and A-CA groups. We initiated cardiopulmonary resuscitation (CPR) 4 min after CA and measured the cerebral tissue oxygenation index (TOI) using near-infrared spectroscopy (NIRS) before, during, and after CPR. In both groups, the TOI was the lowest at 3–4 min after pre-CPR phase initiation (VF-CA group: 3.4 min [2.8–3.9]; A-CA group: 3.2 min [2.9–4.6]; *p* = 0.386). The increase in TOI differed between the groups in the CPR phase (*p* < 0.001); it increased more rapidly in the VF-CA group (16.6 [5.5–32.6] vs. 1.1 [0.6–3.3] %/min; *p* < 0.001). Seven pigs surviving for 60 min after the return of spontaneous circulation in the VF-CA group recovered limb movement, whereas only one in the A-CA group (*p* = 0.023) achieved movement recovery. The increase in the TOI did not differ significantly between the groups in the post-CPR phase (*p* = 0.341). Therefore, it is better to monitor ScO_2_ concomitantly with CPR initiation using NIRS to assess the responsiveness to CPR in clinical settings.

## 1. Introduction

Out-of-hospital cardiac arrest (CA) occurs in approximately 120,000 people in Japan, 350,000–700,000 in Europe, and 330,000 in the United States annually [1,2,3]. Cardiopulmonary resuscitation (CPR) is performed to achieve the return of spontaneous circulation (ROSC) and to improve neurological outcomes. However, only 2% of CA cases in Japan have favourable neurological outcomes [4]. Post-CA neurological damage is mediated by ischaemic injury caused by the interruption of blood flow during CA and reperfusion injury [5,6]. To prevent ischaemic injury due to CA, it is important to assess the amount of oxygen delivered to the brain during CPR compared to that before CPR.

Near-infrared spectroscopy (NIRS) facilitates real-time non-invasive measurement of cerebral oxygen saturation (ScO_2_) during CPR, and NIRS monitoring can predict ROSC and neurological outcomes in patients with CA [7,8,9,10]. However, the use of measurement values obtained during CPR is controversial because few studies have initiated monitoring with NIRS concomitantly with CPR initiation. In most studies, the NIRS monitor was attached while performing CPR [7,8,9,10].

**Figure 1:**
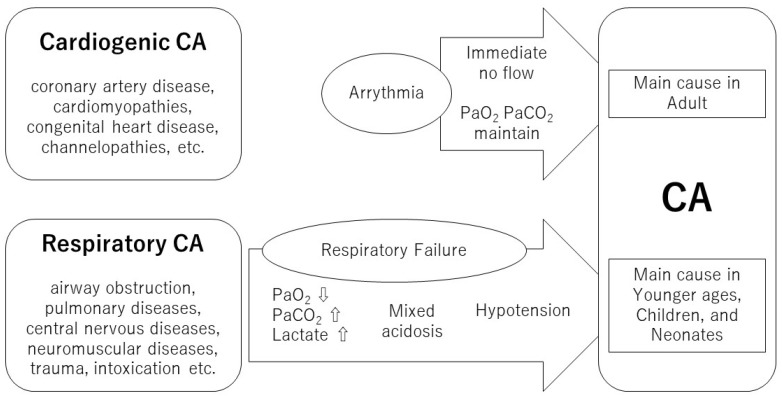
The differences between cardiogenic and respiratory CA. CA, cardiac arrest.

The most prevalent causes of CA in clinical settings are cardiogenic events, followed by respiratory events [4]. Cardiogenic CA is caused by arrhythmia triggered by coronary artery disease, cardiomyopathies, congenital heart disease, and channelopathies, among others [11,12,13,14]. It occurs when the circulation suddenly stops while oxygenation and carbon dioxide levels in the blood are maintained. In contrast, respiratory CA is caused by respiratory failure due to airway obstruction, pulmonary diseases, and neuromuscular causes [15,16]. In the case of respiratory CA, severe hypoxia, elevated partial pressure of carbon dioxide, severe mixed acidosis, and gradual deterioration occur, leading to CA (Figure 1) [17]. Post-CA syndrome presents a similar pathophysiology to acute respiratory distress syndrome. However, a previous report stated that respiratory CA with respiratory failure preceding CA does not differ from cardiogenic CA in the incidence of acute respiratory distress syndrome after resuscitation [18]. Therefore, the exact pathophysiological mechanisms remain unclear. Previous studies have compared the ROSC rate, neurological outcomes, and biomarkers between cardiogenic and respiratory CA events [19,20]. However, to the best of our knowledge, no study has examined the differences in ScO_2_ trends between cardiogenic and respiratory CA events.

Therefore, we aimed to compare changes in ScO_2_ levels during different events in animal models of ventricular fibrillation CA (VF-CA; a cardiogenic event) and asphyxial CA (A-CA; a respiratory event). We hypothesised that ScO_2_ values before CPR similarly decrease immediately after VF induction and asphyxia when cerebral oxygen delivery is impaired and that the ScO_2_ during CPR would be lower and less likely to rise for A-CA than for VF-CA because A-CA takes longer to develop into CA and low ScO_2_ continues. We compared the changes in the ScO_2_ levels before, during, and after CPR between these events. 

## 2. Materials and Methods

All experimental procedures were approved by the Institutional Animal Research Committee of our University (17-359). The animals were cared for in conformance with the National Institute of Health Guidelines for the use and care of animals.

### 2.1. Animal Preparation

Twenty female pigs were randomly categorised into VF-CA and A-CA groups (Table 1). They were intramuscularly administered 500 mg of ketamine as sedation/analgesia and subsequently restrained in the supine position. Intravenous lines were placed on the ear vein of each pig for continuous infusion of propofol (loading concentration, 1 mg/kg; maintenance concentration, 4 mg/kg/h) and vecuronium (loading concentration, 0.6 mg/kg; maintenance concentration, 0.4–0.6 mg/kg/h) [21,22]. 

Under local anaesthesia, using 1.0% lidocaine solution, the animals underwent tracheostomy; a 7-mm inner-diameter endotracheal tube (ETT) was placed in the trachea. The animals were ventilated using LTV-1000 ventilators (CareFusion, San Diego, CA, USA) under the following ventilator settings: tidal volume, 10 mL/kg; positive end-expiratory pressure, 6 cm H_2_O; inspired oxygen fraction, 0.21; and respiratory rate titrated to achieve an end-tidal carbon dioxide (EtCO_2_) value of 40–45 mmHg. A femoral artery catheter was surgically inserted to monitor arterial pressure and collect blood samples. Additionally, a Swan–Ganz catheter (Model 744F8, Edwards Lifesciences, Irvine, CA, USA) was placed through the femoral vein to monitor the right atrial pressure. A 5F-pacing catheter was placed in the opposite femoral vein. We confirmed the positions of the ETT, Swan–Ganz catheter, pacing catheter, and heart positions using a C-arm fluoroscopic X-ray system (DHF-105CX, Hitachi, Tokyo, Japan).

### 2.2. Cerebral Oxygenation

NIRO 200NX^TM^ (Hamamatsu Photonics, Hamamatsu, Japan), a continuous NIRS monitor, was used to measure ScO_2_ (tissue oxygenation index; TOI) using a spatially resolved spectroscopy technique. The TOI was defined as the ratio of oxygenated haemoglobin to total haemoglobin, expressed as an absolute percentage. The normal TOI values were between 50% and 80% [23,24]. In humans, this device is applied to the supraorbital region; it follows an elliptical trajectory approximately 2 cm deep within the cerebral tissue to assess cerebral oxygenation [7]. In this study, two NIRO 200NX^TM^ probes were attached to the intact left and right skin patches covering each cerebral hemisphere anterior to the coronal suture [25]. After completing the experiment, we dissected the brains and confirmed that the distance from the scalp to the brain did not exceed 1.5 cm; therefore, the device could measure cerebral oxygenation in our animal model, as it had a penetration depth of 3 cm.

Because of the limited number of probes, monitoring of brain tissue oxygenation (PtO_2_) using an intracerebral oxygenation probe (Licox^TM^, Integra Life Sciences, Plainsboro, NJ, USA) was possible for only four and three pigs in the VF-CA and A-CA groups, respectively. A 2-mm hole was drilled between the left and right NIRO 200NX^TM^ probes, a 22-gauge cannula was inserted until cerebrospinal fluid outflow was confirmed, and a Licox^TM^ probe was fixed such that it protruded 2 mm from the cannula tip.

### 2.3. Data Collection

Vital signs, including heart rate, heart rhythm, arterial blood pressure, right atrial pressure, respiratory rate, a saturation of percutaneous oxygen (SpO_2_), and EtCO_2_ were monitored using the Philips IntelliVue MP50 patient monitor (Philips Medizin Systeme, Boblingen, Germany). The arterial blood gas (ABG) was measured using the EPOC (Alere, Waltham, MA, USA) or ABL 735 (Radiometer Copenhagen, Copenhagen, Denmark) blood gas analyser. TOI data were obtained from the left and right probes.

### 2.4. Study Design

After calibrating and synchronising all monitoring equipment, Ringer’s solution was rapidly infused, and the animals were stabilised for 30 min to achieve steady vital signs. Subsequently, the baseline TOI, vital signs, and ABGs were recorded. After 10 min, the pre-CPR phase was started.

**Figure 2:**
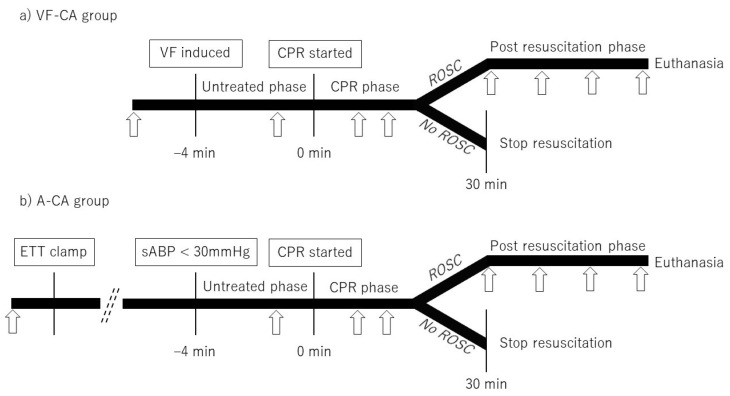
Experimental protocol. Arrows indicate the time points at which arterial blood gas was recorded (at baseline, −1 min, and 3 min after CPR initiation and every 4 min thereafter; immediately after ROSC and every 20 min subsequently). VF-CA, ventricular fibrillation cardiac arrest; VF, ventricular fibrillation; CPR, cardiopulmonary resuscitation; ROSC, return of spontaneous circulation; A-CA, asphyxial cardiac arrest; ETT, endotracheal tube; sABP, systolic arterial blood pressure.

In the VF-CA group, VF was induced using a pacing catheter and an electrical stimulator (GY600A^TM^; Kaifeng Huanan Equipment Co., Ltd., Kaifeng, China), and ventilator support was disengaged. In the VF-CA group, the time of VF induction was set as the CA time. In the A-CA group, asphyxia was induced by clamping the ETT, followed by withdrawal of ventilator support. The CA time was defined as the time when aortic systolic pressure dropped below 30 mmHg [19]. We initiated CPR at 4 min after the onset of CA (Figure 2). During CA, the continuous infusion of propofol and vecuronium was simultaneously stopped in both groups.

In the CPR phase, we standardised the quality of chest compressions using the LUCAS 2^TM^ device (LUCAS^TM^, Jolife, Lund, Sweden). Compressions were localised over the retrosternal position of the heart, which was previously confirmed using fluoroscopy. Simultaneously with CPR initiation, respirator support was restarted at the following ventilatory settings: tidal volume, 10 mL/kg; positive end-expiratory pressure, 6 cm H_2_O; inspired oxygen fraction, 1.0; and respiratory rate, 10 breaths/min. We monitored the pulse and analysed the heart rhythm every 2 min. During the first 2 min of CPR, only basic life support was provided, comprising chest compressions and ventilation asynchronously. Subsequently, if VF persisted, we repeated defibrillation (monophasic, 360 J; Nihon Kohden, Tokyo, Japan) at 2-min intervals and administered adrenaline (1 mg) every 4 min after the first 2 min of defibrillation. Similarly, if the pulseless electrical activity or asystole persisted, adrenaline (1 mg) was administered every 4 min after the first 2 min of CPR as advanced cardiopulmonary life support. We did not use antiarrhythmic drugs as described previously [19].

We stopped CPR in animals that did not achieve ROSC within 30 min of CPR initiation. Animals that achieved ROSC within 30 min continued to receive a rapid infusion of Ringer’s solution (post-CPR phase; Figure 2) and were ventilated at the following settings: tidal volume, 10 mL/kg; positive end-expiratory pressure, 6 cm H_2_O; inspired oxygen fraction adjusted to maintain a SpO_2_ of 93–98%; and a respiratory rate, 10 breaths/min. Surviving animals received no additional medication, including dopamine, phenylephrine, or lidocaine. Independent limb flexion and extension during the post-CPR phase were considered signs of movement function recovery in the subjects; continuous infusion of propofol and vecuronium was restarted in these animals. Those that survived for 60 min after ROSC were euthanised with potassium chloride. We recorded the median ABP and EtCO_2_ values continuously from the pre- to the post-CPR phase, and ABG data were recorded in each phase (Figure 2).

### 2.5. Statistical Analyses

All data are presented as medians (interquartile ranges [IQR]). The TOI value, time, vital signs, and ABG were analysed non-parametrically using the Mann–Whitney *U* test. Yates’ chi-square test was used for intergroup comparisons of the ROSC rate and movement recovery. The TOI was measured repeatedly over time. We used a linear mixed model to account for the longitudinal nature of the data [26,27]. The correlation between the repeated measures recorded for each subject was determined using a random intercept and slope. In case of significant differences, the TOI values obtained every 1 min in the pre-CPR and CPR phases and every 2 min in the post-CPR phase were compared using the Mann–Whitney *U* test. Correlation coefficients of the associations between the TOI and PtO_2_, SaO_2_ (arterial oxygen saturation), and partial pressure of oxygen during ABG recording were determined. The Friedman test (Bonferroni’s multiple comparisons, if significant) was used to identify the strongest correlation.

All tests were two-sided with a significance level of 0.05. All statistical analyses were conducted using the EZR Ver 1.52 (Saitama Medical Centre, Jichi Medical University, Saitama, Japan) [28], which is a graphical user interface for R software (The R Foundation for Statistical Computing, Vienna, Austria).

## 3. Results

### 3.1. Baseline

None of the animals experienced CA during preparation. The vital signs and ABG values did not differ significantly between the VF-CA and A-CA groups (Table 1). We used 280 (200–320) and 155 (125–205) mg propofol (*p* = 0.071); 11.0 (8.0–14.0) and 4.0 (3.4–5.5) mg vecuronium (*p* = 0.009); and 400 (250–500) and 300 (238–300) mL Ringer’s solution (*p* = 0.352) in the VF-CA and A-CA groups, respectively. Propofol and vecuronium doses were higher in the VF-CA group because the number of cases that needed drugs for VF induction before or after ROSC was higher in this group.

### 3.2. Pre-CPR Phase

**Figure 3:**
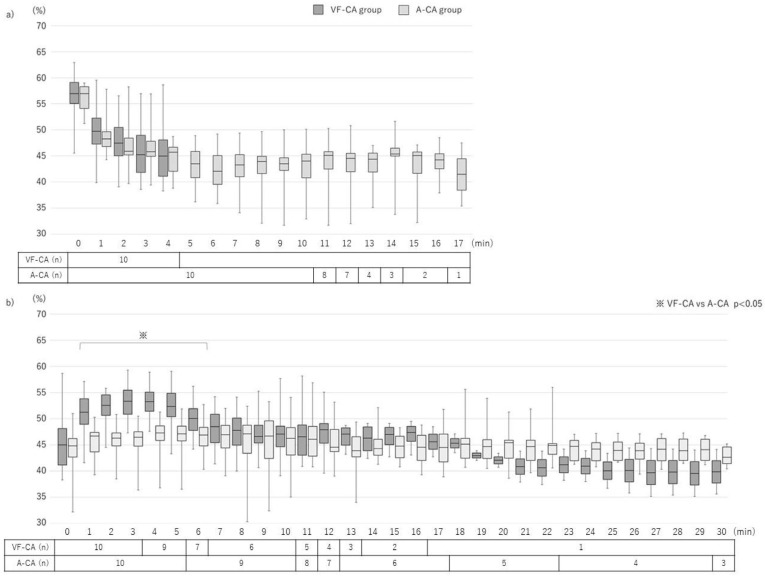
Changes in cerebral oxygen saturation (tissue oxygenation index; TOI) in the pre-CPR and CPR phases. (**a**) Pre-CPR phase: 4 min elapsed from VF induction to CPR initiation in the VF-CA group; 13.9 (12.5–15.3) min elapsed from clamping to CPR initiation in the A-CA group. The TOI increased comparably in both groups (*p* = 0.188). (**b**) CPR phase: the TOI values increased differently in both groups (*p* < 0.001). Between 1 and 6 min after CPR initiation, the TOI was higher in the VF-CA group than in the A-CA group. Values are presented as medians (interquartile ranges). VF-CA, ventricular fibrillation cardiac arrest; A-CA, asphyxial cardiac arrest; CPR, cardiopulmonary resuscitation.

In the A-CA group, the interval between clamping and CA onset was 9.9 (8.5–11.3) min. The TOI reached the minimum value at 3–4 min after initiation of the pre-CPR phase in both groups (VF-CA group, 3.4 [2.8–3.9] min; A-CA group, 3.2 [2.9–4.6] min; *p* = 0.386; Table 2). TOI decrease within the 4-min interval after VF induction and clamping did not differ significantly (*p* = 0.188; Figure 3a). The interval between the lowest TOI value and CPR initiation was longer in the A-CA group than in the VF-CA group (10.0 min [8.6–12.1] vs. 0.6 [0.1–1.2] min; *p* < 0.001).

### 3.3. CPR Phase

In total, nine (90%) and six (60%) subjects achieved ROSC in the VF-CA and A-CA groups, respectively (*p* = 0.303; Table 3). CPR duration did not differ significantly between the two groups (VF-CA group, 10.5 [5.3–12.8] min; A-CA group, 19.5 [11.3–29.8] min; *p* = 0.085). The initial and median values of the mean arterial blood pressure (mABP) were significantly higher in the VF-CA group than in the A-CA group (Table 2). The increase in the TOI was significantly different between the groups (*p* < 0.001). In both groups, the TOI value increased rapidly up to a certain value and gradually increased thereafter (Figure 3b). The slope at every 5-s interval up to the maximum TOI value was measured, and the peak of the slope was defined as the singular point. The TOI velocity from CPR initiation to the singular point was higher in the VF-CA group than in the A-CA group (16.6 [5.5–32.6] %/min vs. 1.1 [0.6–3.3] %/min; *p* < 0.001). The time from CPR initiation to the singular point and to reach the maximum TOI value was shorter in the VF-CA group than in the A-CA group (0.3 [0.2–0.4] min vs. 1.2 [0.5–4.6] min; *p* = 0.002, 3.1 [1.8–4.3] min vs. 6.5 [3.8–10.3] min; *p* = 0.003). 

The maximum and median TOI values were higher in the VF-CA group than in the A-CA group (Table 2). The initial TOI values did not differ significantly between the two groups at CPR initiation. In contrast, at 1–6 min after CPR initiation, the TOI values were higher in the VF-CA group than in the A-CA group (Figure 3b).

### 3.4. Post-CPR Phase

In total, seven (70%) and three (30%) animals survived for 60 min after ROSC in the VF-CA and A-CA groups, respectively (*p* = 0.180; Table 3). All seven animals in the VF-CA group recovered movement, whereas only one pig in the A-CA group showed movement recovery (*p* = 0.023; Table 3). Survival time did not differ significantly between the two groups (VF-CA, 60.0 [59.0–60.0] min; A-CA, 57.5 [25.3–59.8] min; *p* = 0.335). 

**Figure 4:**
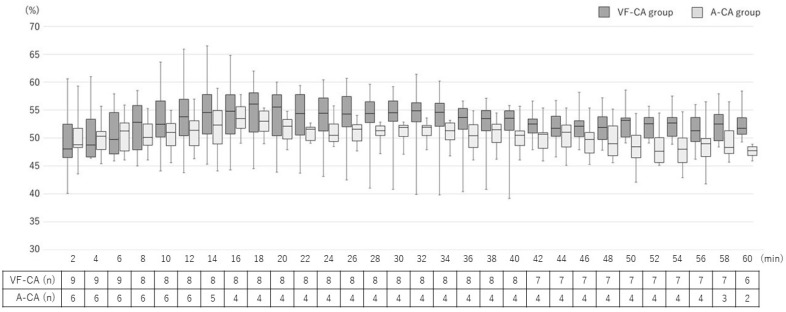
Changes in cerebral oxygen saturation (tissue oxygenation index; TOI) in the post-CPR phase. The increase in the TOI was similar between the groups (*p* = 0.180). VF-CA, ventricular fibrillation cardiac arrest; A-CA, asphyxial cardiac arrest; TOI, tissue oxygenation index; CPR, cardiopulmonary resuscitation.

The increase in TOI did not significantly differ between the two groups (*p* = 0.341); the TOI increased slowly and then plateaued in both groups (Figure 4). The interval from ROSC to the maximum TOI did not differ significantly between the two groups (VF-CA, 16.3 [5.3–29.1] min; A-CA, 8.1 [2.8–11.2] min; *p* = 0.072).

### 3.5. Correlation between the TOI and Cerebral Oxygenation

Correlation coefficients of the association of the TOI with PtO_2_, SaO_2_, and PaO_2_ (partial pressure of arterial oxygen) were 0.800 [0.637–0.95], 0.404 [0.335–0.466], and 0.201 [−0.071 to 0.305], respectively. PtO_2_ displayed the strongest correlation with the TOI (*p* < 0.001; PtO_2_ vs. SaO_2_, *p* = 0.002; PtO_2_ vs. PaO_2_, *p* < 0.001; SaO_2_ vs. PaO_2_, *p* < 0.001).

## 4. Discussion

Here, the ScO_2_ values reached a trough at approximately 4 min after VF induction and clamping in the VF-CA and A-CA groups, respectively. The ScO_2_ values in the A-CA group remained low from 4 min after clamping until CPR initiation. Furthermore, after CPR initiation, the ScO_2_ values increased more rapidly in the VF-CA group than in the A-CA group.

During a CA event, oxygen levels decline in the brain, followed by membrane pump arrest within 3–5 min of complete ischaemic anoxia [6,29] and the initiation of hypoxia-induced irreversible changes. The PtO_2_ and ScO_2_ reach their lowest values at 4 min after VF induction [25,30]. The ScO_2_ reflects changes in both the delivery and consumption of oxygen in the brain [10]. Brain oxygen saturation is correlated with partial pressure of oxygen in the brain tissue [25,31]. In the VF-CA group, the ScO_2_ values decreased because circulation abruptly stopped and oxygen could not be delivered to the brain. Conversely, although the ScO_2_ reached its lowest value in a similar time duration after clamping in the A-CA group, ScO_2_ values persisted at low levels because oxygen could not be delivered to the brain despite continued circulation. Therefore, the ScO_2_ decreased when oxygen could not be delivered to the brain and was minimised at 4 min after anoxia started developing in the brain in both the VF-CA and A-CA groups. 

In the pre-CPR phase (1 min before CPR), the ABG results of the A-CA group were worse than those of the VF-CA group. In the VF-CA group, ABG was measured at 3 min after circulation abruptly stopped while oxygenation was maintained; therefore, the PaO_2_ and partial pressure of arterial carbon dioxide were maintained, and the lactate levels did not increase. In contrast, in the A-CA group, ABG was measured at approximately 13 min after clamping (oxygen could not be delivered suddenly, and carbon dioxide could not be discharged; however, circulation was maintained for approximately 10 min). Therefore, lactic acid was produced under anaerobic conditions, and acidosis worsened [32].

The ROSC rate was 90–100% and 60% in the VF-CA and A-CA groups, respectively, during CPR initiation at 8 min after CA onset [19,20]. Moreover, the mABP after ROSC was lower in the A-CA group, causing hypoxia-induced cardiac dysfunction [20]. Here, although we initiated CPR at 4 min after the onset of CA, the ROSC rates observed were consistent with those reported previously [19,20]. The mABP values during CPR and after ROSC were lower in the A-CA group than in the VF-CA group. In the VF-CA group, the median ScO_2_ value was >50% during CPR, and the maximum ScO_2_ value was achieved approximately 3 min after CPR initiation. Conversely, in the A-CA group, the median ScO_2_ value remained <50% during CPR, and the maximum ScO_2_ value was achieved at 6.5 min after CPR initiation. Furthermore, the ScO_2_ values were significantly higher in the VF-CA group than in the A-CA group at 1–6 min after CPR initiation because the VF-CA group had a short duration of anoxia with low ScO_2_ values, consistent mABP of >65 mmHg following CPR initiation, and immediate oxygen delivery to the brain, resulting in a rapid increase in the ScO_2_ values with good responsiveness to CPR. In contrast, the A-CA group had long-duration anoxia with low ScO_2_ values and low mABP at CPR initiation, which hampered immediate oxygen delivery to the brain and resulted in the gradual increase of ScO_2_ with poor responsiveness to CPR.

The degrees of hypoxia and ischaemia considerably increased after asphyxia than after VF [33]. NIRS monitoring facilitates the understanding of the degree of hypoxia before and during CPR. However, in many clinical settings, the NIRS monitor was attached during CPR; thus, it remains unclear whether the initial ScO_2_ value immediately after attaching the monitor and the maximum, minimum, or average values during CPR should be used as indicators for ROSC and neurological outcomes [34]. Moreover, congenital heart disease, which is a cause of cardiogenic CA, may cause low ScO_2_, depending on the heart or vascular anomaly; therefore, a low ScO_2_ value may not be associated with poor outcomes in some diseases [13]. This study showed that the timepoint of the initiation of NIRS monitoring might obscure the significance of the differences in the ScO_2_ values between VF-CA and A-CA events. During NIRS monitoring in a pre-hospital setting, the values obtained after NIRS monitor attachment at CPR initiation revealed an initial ScO_2_ value of approximately 30% in both the ROSC and non-ROSC groups [26]. In our study, both groups displayed a minimum ScO_2_ value of approximately 40% at CPR initiation. Measuring the ScO_2_ on the NIRS monitor from CPR initiation is better for assessing responsiveness and predicting the ROSC, neurological outcomes, and cause of CA. Nevertheless, further studies are needed to determine whether NIRS data can predict ROSC, neurological outcomes, and the cause of CA.

### Limitations

This study had some limitations. First, our observations were restricted to a 60-min time duration after ROSC. The lack of adequate vital signs after ROSC indicated the possibility of organ damage before ROSC, which may have affected the neurological outcomes. Furthermore, limb movement within 1 h alone is insufficient to assess neurologic function. However, limb movement is the criterion for stopping CPR in basic life support, and the presence of limb movement is important for good neurological outcomes.

Second, one pig in the VF-CA group did not achieve ROSC with 10 defibrillations. If defibrillation is not successful early, the duration of CPR is prolonged, the mABP is lowered, oxygen delivery is worsened, and the ScO_2_ decreases. Combined with cardiac dysfunction, these factors make it impossible to achieve ROSC. Conversely, successful defibrillation in the presence of consistently high ScO_2_ values may be an important factor during CPR. Only one animal in the A-CA group recovered independent movement. At approximately 7 min after clamping, this animal experienced VF and CA (shortest time to CA in the A-CA group). The short durations in the low ScO_2_ condition may lead to favourable neurological outcomes. 

Third, the small sample size did not allow parametric statistical analysis. Fourth, the lower mABP could be due to asphyxia injury during the longer pre-CPR phase in the A-CA group. This could lower cerebral perfusion pressure and reduce oxygen delivery. Fifth, the effect of sedatives and analgesics on the ROSC rate and movement recovery cannot be denied. However, the VF-CA group had better ROSC and movement recovery rates than the A-CA group, even though it took longer to induce VF in that group and more medications were required.

Finally, we started CPR at 4 min after the onset of CA, which is when hypoxia-induced irreversible changes are initiated. A shorter time after the onset of CA would have resulted in more pronounced differences between the groups because the ScO_2_ would have increased immediately with a smaller decrease in the VF-CA group. Conversely, the ScO_2_ changes in subjects with long durations of CA (even cardiogenic) remain unknown. Therefore, further studies are needed to confirm the findings of this study.

## 5. Conclusions

The ScO_2_ values reached a trough at approximately 4 min after VF induction and clamping in the VF-CA and A-CA groups, respectively. Furthermore, the ScO_2_ value increased more rapidly after CPR initiation in the VF-CA group than in the A-CA group. Importantly, it is better to initiate ScO_2_ monitoring using NIRS concomitantly with CPR initiation to assess the responsiveness to CPR.

## Figures and Tables

**Table 1:** Baseline characteristics of the subjects.

	VF-CA (N = 10)	A-CA (N = 10)	*p*-Value
Age (days)	67.5 (60.3–71.0)	76.0 (61.5–81.5)	0.201
Body weight (kg)	24.0 (23.8–25.2)	23.3 (22.2–23.8)	0.055
TOI (%)	58.2 (55.1–60.1)	56.3 (55.4–57.4)	0.069
**Vital signs**			
mABP (mmHg)	103.5 (92.0–113.5)	80.0 (69.3–94.8)	0.134
EtCO_2_ (mmHg)	42.5 (40.5–46.0)	43.5 (40.5–46.5)	0.573
**Arterial blood gas**			
pH	7.43 (7.41–7.47)	7.43 (7.41–7.46)	0.573
P_a_O_2_ (mmHg)	93 (76–118)	104 (90–119)	0.416
P_a_CO_2_ (mmHg)	40.9 (38.1–44.3)	41.6 (37.7–43.7)	0.564
Lactate (mmol/L)	2.70 (1.65–3.58)	2.54 (1.82–4.38)	0.535

Data are presented as medians (interquartile ranges). VF-CA, ventricular fibrillation cardiac arrest; A-CA, asphyxial cardiac arrest; TOI, tissue oxygenation index; mABP, mean arterial blood pressure; EtCO_2_, end-tidal CO_2_; P_a_CO_2_, partial pressure of arterial carbon dioxide; P_a_O_2_, partial pressure of arterial oxygen.

**Table 2:** Comparison of the ventricular fibrillation cardiac arrest (VF-CA) and asphyxial cardiac arrest (A-CA) groups.

	Pre-CPR Phase		CPR Phase		Post-CPR Phase	
	VF-CA	A-CA	VF-CA	A-CA	VF-CA	A-CA
**TOI (%)**						
Initial	58.2 (55.1–60.1)	56.3 * (55.4–57.4)	45.0 (41.2–48.2)	44.9 (42.7–46.2)	50.1 (46.2–54.6)	49.2 (47.2–51.2)
Maximum	58.8 (55.4–60.5)	56.9 (55.6–57.8)	56.8 (52.6–59.4)	49.0 * (47.9–50.6)	59.0 (56.4–61.5)	53.1 * (51.3–56.5)
Minimum	41.8 (40.6–46.9)	39.3 * (35.6–41.4)	41.9 (39.2–43.4)	42.5 (38.4–44.3)	45.6 (41.9–47.8)	45.1 (42.9–46.8)
Median	48.5 (45.1–50.2)	45.1 * (43.2–45.4)	49.3 (46.3–53.4)	46.3 * (44.6–48.8)	52.0 (49.9–54.3)	49.6 (47.8–52.0)
Value at the singular point	–	–	50.1 (48.7–52.5)	47.2 * (44.9–48.2)	–	–
**Vital signs (mmHg)**						
mABP initial	–	–	69.5 (60.0–88.0)	30.5 * (17.8–39.0)	143.5 (113.8–167.8)	108.5 (45.3–127.5)
mABP median	30.0 (23.0–32.8)	26.0 (15.5–28.8)	66.3 (58.1–78.5)	50.0 * (44.0–57.3)	62.0 (57.0–84.0)	56.0 (51.0–63.3)
EtCO_2_ median	–	–	22.0 (17.5–31.0)	22.5 (11.8–34.6)	38.0 (35.0–41.0)	37.0 (25.8–40.8)
**Arterial** **b** **lood gas**						
pH	7.42 (7.41–7.45)	7.01 * (6.95–7.03)	7.299 (7.212–7.458)	7.141 * (7.074–7.219)	7.171 (7.125–7.221)	6.980 (6.926–7.107)
P_a_O_2_ (mmHg)	76.8 (56.7–96.6)	10.2 * (5.8–12.5)	137.0 (109.0–202.8)	165.8 (71.8–219.2)	166.0 (121.0–265.0)	259.7 (215.1–368.5)
P_a_CO_2_ (mmHg)	42.5 (37.7–46.6)	107.0 * (100.2–131.3)	50.8 (31.5–54.4)	48.7 (33.7–56.9)	53.0 (38.9–56.2)	46.9 (43.5–57.0)
Lactate (mmol/L)	2.7 (1.8–3.6)	12.0 * (7.8–14.2)	8.1 (7.3–8.9)	14.4 * (13.3–15.5)	10.1 (9.1–12.9)	15.2 * (14.2–18.2)

Data are presented as medians (interquartile ranges). * The values for the A-CA group were significantly different from those for the VF-CA group in each phase (*p* < 0.05). CPR, cardiopulmonary resuscitation; TOI, tissue oxygenation index; VF, ventricular fibrillation; ROSC, return of spontaneous circulation; mABP, mean arterial blood pressure; EtCO_2_, end-tidal CO_2_.

**Table 3:** Outcomes of subjects in the ventricular fibrillation cardiac arrest (VF-CA) and asphyxial cardiac arrest (A-CA) groups.

	VF-CA (*n* = 10)	A-CA (*n* = 10)	*p*-Value
ROSC	9	6	0.303
Survived for 60 min after ROSC	7	3	0.180
Recovered movement	7	1	0.023

ROSC, return of spontaneous circulation.

## Data Availability

The data that support the findings of this study are available from the corresponding author, Y.K., upon reasonable request.
